# Experiments in Re-vaccination

**Published:** 1857-06

**Authors:** 


### ECLECTIC DEPARTMENT,

### AND SPIRIT OF THE MEDICAL PERIODICAL PRESS.

---

The following statement on re-vaccination, are interesting:

*Experiments in Re-vaccination.*—We find in a late number of the *Zeitschrift der k. k. Gesellschaft der Aertzte zu Vien*, the following results of re-vaccination in the House of Correction, at Vienna, by Dr. F. Scholz. The trials were made on 126 men and 37 women, and the re-vaccination succeeded in 36 men and 12 women. The following were the conclusions of the writer:

1. The average proportion of successful cases was one in three.
2. The sex of the patient exercised no influence on the result.
3. The susceptibility to re-vaccination was in the inverse proportion to the number and perfection of the cicatrices left by previous vaccination.
4. This susceptibility increased in proportion to the length of time between the vaccination and the re-vaccination.
5. The increased susceptibility to the vaccine disease manifested itself both in the greater number of cases, and the greater number of perfect vesicles in each case.
6. In a few cases, variolous disease occurred 11 years after previous vaccination, and in no case did re-vaccination succeed earlier than eight years after variolous disease.
7. In no case was the normal course of revaccination accompanied by febrile symptoms, and when these did occur, the patient was either suffering from other disease, or the course of the vaccine disease was abnormal.
8. The majority of chronic diseases were not modified by re-vaccination, nor did they exercise any particular influence on the course of the vaccine vesicle.
9 In two cases occompanied by chronic spasms were the convulsions suspended during the course of the vaccine disease.—*Boston Med. Journal.*

